# Acute hepatocellular and cholestatic injury during therapy with hydrochlorothiazide - clinicohistopathologic findings: a case report

**DOI:** 10.1186/1752-1947-4-332

**Published:** 2010-10-21

**Authors:** Fabrizio Taglietti, Franca Del Nonno, Andrea Baiocchini, Laura Falasca, Stefano Pieri, Alessandro Capone, Elisabetta Grilli, Pierangelo Chinello, Nicola Petrosillo

**Affiliations:** 1 II Division of Infectious Diseases, Istituto Nazionale per le Malattie Infettive, Istituto di Ricovero e Cura a Carattere Scientifico 'Lazzaro Spallanzani', Rome, Italy; 2 Department of Pathology, Istituto Nazionale per le Malattie Infettiv, Istituto di Ricovero e Cura a Carattere Scientifico 'Lazzaro Spallanzani', Rome, Italy; 3 Laboratory of Electron Microscopy, Istituto Nazionale per le Malattie Infettive, Istituto di Ricovero e Cura a Carattere Scientifico 'Lazzaro Spallanzani', Rome, Italy; 4 Radiology Department, San Camillo-Forlanini Hospital, Rome, Italy

## Abstract

**Introduction:**

Hydrochlorothiazide and thiazide-like diuretics are considered first-line drugs for initial therapy in uncomplicated arterial hypertension. Acute cholecystitis is a well-known complication during treatment with thiazide, and these drugs are also reported to be followed by pronounced insulin resistance.

**Case presentation:**

We describe a case of acute cholestatic hepatitis in a 68-year-old Caucasian man who was receiving olmesartan and hydrochlorothiazide for arterial hypertension. From the clinical and histologic findings, we diagnosed him as having hepatocellular-cholestatic injury and a disorder of glucose metabolism in the liver. To the best of our knowledge, no histopathologic description of hydrochlorothiazide hepatotoxicity has previously been documented in the literature.

**Conclusion:**

In the differential diagnosis of cholestatic hepatitis, clinicians should be aware of the possibility of liver damage in patients receiving hydrochlorothiazide therapy.

## Introduction

Thiazide diuretics are first-line and low-cost drugs used to treat uncomplicated arterial hypertension [1]. They were originally synthesized in an effort to enhance the potency of inhibitors of carbonic anhydrase. However, unlike carbonic anhydrase inhibitors, which primarily increase Sodium Bicarbonate (NaHCO_3) _excretion, thiazides were found predominantly to increase Sodium Chloride (NaCl) excretion, an effect shown to be independent of carbonic anhydrase inhibition.

The major concerns about their use arise from their tendency to cause hypokalemia, impair glucose tolerance, and increase serum cholesterol and uric acid. Similar to loop diuretics, the most serious adverse events are related to abnormalities of fluid and electrolyte balance [2].

The most common adverse events of thiazide diuretics include vertigo, headache, paresthesias, xanthopsia, weakness, anorexia, nausea, vomiting, cramping, diarrhea, constipation, cholecystitis, pancreatitis, blood dyscrasias, photosensitivity, and skin rashes [2-7].

Thiazide diuretics also decrease glucose tolerance, and latent diabetes mellitus may be unmasked during therapy. The mechanism behind the impaired glucose tolerance is not completely understood, but appears to involve reduced insulin secretion and alterations in glucose metabolism [8].

Thiazide diuretics also may increase plasma levels of low-density lipoprotein cholesterol, total cholesterol, and total triglycerides. Hepatotoxicity by hydrochlorothiazide (HCTZ) therapy is an uncommonly adverse event rarely described, and only clinically [9].

We describe a clinical case of HCTZ-induced acute cholestatic hepatitis associated to alterations of the glucose metabolism inside the liver.

## Case presentation

A 68-year-old Caucasian man was admitted to our Infectious Diseases Unit with a ten-day history of jaundice, asthenia, nausea, vague right upper quadrant abdominal pain and hyperchromic urine. Twenty days previously, while on treatment with ramipril, the patient consulted his general practitioner because of recent onset of high systolic arterial pressure value. Treatment with ramipril was stopped, and olmesartan 10 mg and HCTZ 12.5 mg were started. At this time, our patient's clinical and routine laboratory findings were normal.

On admission to our hospital, the patient appeared asthenic. On physical examination, we found the patient to have a normal body mass index, vague right upper quadrant abdominal pain, sclera icterus and mild hepatomegaly. The remainder of the clinical examination was unremarkable. Other than the arterial hypertension, he had no medical history of note.

Liver function test results showed: alanine aminotransferase (ALT) 346 U/L (normal < 40 U/L), aspartate aminotransferase (AST) 158 U/L (< 40 U/L), gamma glutamine transferase (GGT) 250 U/L (< 64 U/L), C-reactive protein 0.70 mg/dL (< 0.60 mg/dL), alkaline phosphatase 1.091 U/L (normal range 91 to 258 U/L), total bilirubin 6.28 mg/dL (0.2 to 1 mg/dL), direct bilirubin 4.26 mg/dL (0.01 to 0.2 mg/dL), ferritin 647 ng/dL (15 to 400 ng/dL). Results of red and white blood cell counts, platelet counts, and other laboratory tests were normal.

Results of tests for possible infectious causes of hepatitis showed that our patient was negative for cytomegalovirus (CMV) IgM, parvovirus B19 IgM and IgG, and Epstein-Barr viral capsid antigen (EBV VCA) IgM, and positive for CMV IgG and EBV VCA IgG positive. Blood cultures, and antibody tests for human immunodeficiency virus, hepatitis A, B, C and E viruses, hepatitis surface antigen, and leptospira were negative. Our patient's immunoglobulin levels were normal. Antinuclear autoantibody, anti-microsomal antibodies, anti-smooth muscle antibodies, anti-liver-kidney-microsomal antibodies, anti-neutrophil cytoplasmic antibody, and anti-glomerular basement membrane were undetectable. Screening for tumor-associated antigens was also negative.

Abdominal untrasonography, abdomen computed tomography, and magnetic resonance cholangiography were performed to assess the presence of solid tumors or bile duct obstruction; the results revealed only a mild hepatomegaly.

By day 18, our patient was still jaundiced and had scleral icterus. His clinical condition was unchanged. Repeat laboratory investigations showed an increase in total bilirubin (14.7 mg/dL), with direct bilirubin at 12.6 mg/dL. AST and ALT values were slightly diminished (100 and 170 U/L respectively), and alkaline phosphatase and GGT had decreased to 428 U/L and 170 U/L, respectively (Table 1).

**Table 1:** Trend of liver function tests

Blood test	Day				
	Day 0	18^1^	22	30	90
ALT (U/L)^2^	346	170	100	76	20
AST (U/L)^2^	158	100	96	58	14
Total bilirubin (mg/dL)	6.28	14.7	10.6	6	0.2
Direct bilirubin (mg/dL)	4.26	12.6	8.4	4.1	0.1
Alkaline phosphatase (U/L)	1.09	428	340	298	146
GGT (U/L)^2^	250	170	140	98	12

^1^HCTZ therapy stopped. ^2^ALT = alanine amino-transferase; AST = aspartate amino-transferase; GGT = gamma glutammin transferase

Because of our patient's persistent higher transaminase and bilirubin values, and the negative findings on laboratory and radiologic testing, the clinical picture was attributed to primary toxic damage by HCTZ. Therapy with olmesartan plus HCTZ was then stopped; enalapril 10 mg twice daily was started. Using percutaneous needle biopsy, a liver sample was obtained, fixed in 10% buffered formalin, and embedded in paraffin wax for routine histologic examination. Slides were stained with hematoxylin and eosin, periodic acid-Schiff (PAS), PAS diastase, Perl, reticulin silver impregnation, and Masson trichrome.

**Figure 1:**
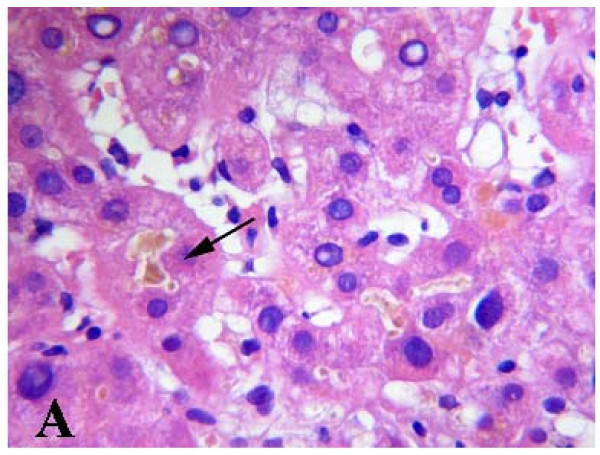
**Marked cytoplasmic canalicular cholestasis (hematoxylin and eosin)**.

**Figure 2:**
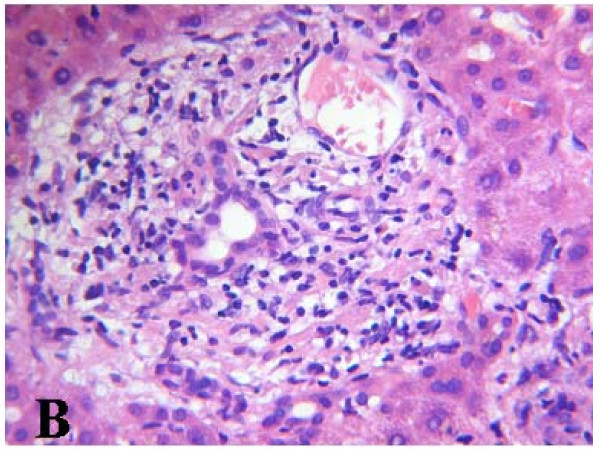
**Mild portal tract edema with a light mixed inflammatory cell infiltrate and periportal hyperglycogenated nuclei (hematoxylin and eosin)**.

Histologic evaluation of the liver sample was performed by two pathologists. There was evidence of acute cholestatic hepatitis. The perivenular hepatocytes were swollen, with feathery degeneration. Bile thrombi in dilated canaliculi (canalicular cholestasis) were also seen throughout the acinus (Figure 1). Doubling of the hepatic trabeculae with anisokaryosis and formation of several liver cell rosettes, features that were most apparent in zone three, were considered features of regeneration. A mild hepatic necroinflammatory mixed infiltrate was present in all three zones of the acinus, in the form of spotty necrosis and occasionally confluent necrosis. There was accumulation and enlargement of Kupffer cells, many of them forming discrete clumps in zone three. They contained yellow-brown ceroid pigment, staining with PAS after diastase digestion. The portal tracts were expanded and slightly oedematous, and contained a moderate inflammatory cell infiltrate composed of lymphocytes and neutrophils, with many eosinophils and histiocytes. Focal interface hepatitis was present in some portal tracts. The interlobular bile ducts were preserved, and ductular reaction was evident at the edge of portal tracts. Zone one hepatocytes were swollen, and staining was pale. Glycogenated nuclei were present in periportal hepatocytes (Figure 2). Finally, granules of stainable iron were seen in sinusoidal lining cells and in periportal hepatocytes, associated with a more diffuse staining indicative of ferritin deposits. Fatty changes and Mallory-Denk bodies were not seen. Mild pericellular fibrosis was present in perivenular areas.

By day 22, our patient's transaminase and bilirubin values were decreased and his clinical condition improved. The glycemic index and insulin index were normal. On day 30, the patient was discharged in good clinical condition. His final blood results were: ALT 76 U/L, AST 58 U/L, alkaline phosphatase 298 U/L, total bilirubin 6.0 mg/dL, direct bilirubin 4.1 mg/dL, GGT 98 U/L and C-reactive protein 0.10 mg/dL. At follow-up examination on day 90, our patient was asymptomatic and his liver blood tests gave normal results. The pattern of blood test results is shown in Table 1.

## Discussion

Cholestatic hepatitis has previously been reported in one patient during HCTZ treatment [9]. In that paper, the diagnosis of HCTZ-induced cholestatic liver injury was based on the laboratory tests and the Naranjo probability scale, indicating a possible relationship between the drug and liver injury. However, to date no histopathological description of the associated liver changes has been reported, to the best of our knowledge, and our case represents the first documentation of clinical and histopathological liver damage in the course of HCTZ treatment.

In our patient we excluded an obstruction of the biliary tract (for example, hepatic and pancreatic neoplasm, biliary calculosis), primitive biliary cirrhosis, sclerosing cholangitis, autoimmune disorders of the liver and infective causes for the increased transaminase values, including viral hepatitis. Based on our patient's history and on his score on the Naranjo probability scale, which indicated a probable relationship between HCTZ and development of liver injury [10], we suspected that his hepatic damage was probably related to the HCTZ therapy. To confirm our hypothesis, we examined a liver biopsy, which showed acute hepatocellular-cholestatic injury associated with alterations of glucose metabolism in the liver, which was consistent with our clinical hypothesis of liver toxicity by HCTZ.

Because a recent article [11] had demonstrated how visceral fat redistribution, liver fat accumulation, low-grade inflammation, and aggravated insulin resistance are all adverse effects caused by HCTZ therapy, we plotted a glycemic index and an insulin index for our patient, which gave normal results. A possible explanation for this was the concomitant treatment with olmesartan, which may prevent hyperglycemia [12]. In addition, the HCTZ treatment duration was relatively short (40 days), possibly too short to cause marked alteration of glucose metabolism, and our patient had no known risk factors for developing diabetes.

## Conclusion

In conclusion, in the differential diagnosis of cholestatic hepatitis, clinicians should be aware of the possibility of liver damage in the course of HCTZ therapy.

## Competing interests

The authors declare that they have no competing interests.

## Consent

Written informed consent was obtained from the patient for publication of this case report and accompanying images. A copy of the written consent is available for review by the Editor-in-Chief of this journal.

## Authors' contributions

FT monitored the patient during hospitalization, analyzed data from the literature, and helped to write the article. FDN, AB and LF performed the histologic examination of the liver biopsy. ST performed the liver biopsy. EG was the major contributor in writing the manuscript. AC and PC performed the follow-up consultations of the patient after discharge. NP reviewed the manuscript. All authors read and approved the final manuscript
